# A Unique Patient Stratification Method Combined with a Machine Learning Approach Identifies Novel Genetic Susceptibility and Protective Factors for Severe COVID-19 in a Hungarian Population

**DOI:** 10.3390/ijms27052358

**Published:** 2026-03-03

**Authors:** Alexandra Neller, Mátyás Bukva, Bence Gálik, József Kun, Nikoletta Nagy, Ferenc Somogyvári, Valéria Endrész, Margit Pál, Barbara Anna Bokor, Zsófia Blazovich, Ádám Visnyovszky, Balázs Bende, Péter Urbán, Szilvia Kovácsné Levang, Zoltán Péterfi, Gábor L. Kovács, Katalin Gombos, Attila Gyenesei, Márta Széll

**Affiliations:** 1 Department of Medical Genetics, University of Szeged, 6720 Szeged, Hungary; neller.alexandra@o365.u-szeged.hu (A.N.); nagy.nikoletta@med.u-szeged.hu (N.N.);; 2 Biological Research Centre Szeged, 6726 Szeged, Hungary; 3 Hungarian Centre for Genomics and Bioinformatics, Szentágothai Research Centre, University of Pécs, 7624 Pécs, Hungary; 4 Department of Pharmacology and Pharmacotherapy, Medical School, University of Pécs, 7624 Pécs, Hungary; 5 Department of Medical Microbiology, University of Szeged, 6725 Szeged, Hungaryendresz.valeria@med.u-szeged.hu (V.E.); 6 Department of Dermatology and Allergology, University of Szeged, 6720 Szeged, Hungary; 7 Clinical Centre, First Department of Internal Medicine, University of Pécs, 6724 Pécs, Hungary; 8 Department of Laboratory Medicine, Medical School, University of Pécs, 7624 Pécs, Hungary; 9 Hungarian National Laboratory on Reproduction, University of Pécs, 7624 Pécs, Hungary

**Keywords:** COVID-19, immunogenetics, machine learning, genomics

## Abstract

Intensive research has shown that severe COVID-19 outcomes are influenced by antiviral pathways and immune responses, both shaped by genetic predisposition. In this study, we aimed to identify genetic variants associated with disease severity in a cohort of Hungarian patients. We applied a novel stratification method based on age, disease severity, and clinical background to classify patients by susceptibility to severe COVID-19. Whole-exome sequencing (WES) was performed on 168 individuals, and gene mutation loads were assessed. Using a Random Forest machine learning approach, we identified variants of 877 genes that distinguished between severe and non-severe cases. We further categorized these genes as either susceptibility or protective factors. Gene-set enrichment analysis highlighted the most affected biological pathways. Our findings support the development of personalized diagnostic tools to assess the risk of severe COVID-19 and guide targeted treatment strategies. Our findings further extend the results of previous studies, providing novel insights into the genetic determinants of COVID-19 severity.

## 1. Introduction

Coronavirus 2019 (COVID-19) disease develops as a consequence of infection with severe acute respiratory syndrome coronavirus 2 (SARS-CoV-2) [1]. Since its emergence in 2019, SARS-CoV-2 has infected hundreds of millions of people worldwide and caused more than seven million deaths as of 10 September 2025 (https://covid19.who.int/, accessed on 10 September 2025). The mortality rate for vulnerable individuals with advanced age and/or medical comorbidities has been notably high. Infected patients exhibit a wide spectrum of COVID-19 severity, ranging from asymptomatic to critically affected. The frequency of asymptomatic infections is estimated to range between 10% and 70%, depending on study design and population characteristics. The disease outcome is highly influenced by multiple factors, such as age, gender, social status, and geographic location [2]. Among symptomatic cases, ~40% present with mild upper respiratory tract infection, ~20% with pneumonia, and ~3% progress to severe disease [3]. Patients of advanced age and those with major comorbidities are more frequently affected by severe and critical COVID-19 development, necessitating admission to intensive care units [4]. The most relevant comorbidities include pulmonary diseases, hypertension, diabetes, obesity, and secondary immunosuppression caused by malignant diseases or medications [5]. Despite these general observations, the social and economic differences between countries make it difficult to create a coherent system for the assessment of the risk factors for the disease. In addition to the typical scenarios described above, COVID-19 can result in other, anomalous outcomes [5]. Anomalies in mortality and morbidity have been observed both between and within populations [6].

SARS-CoV-2 strains that emerged during the pandemic exhibited variability in virulence and COVID-19 severity [7,8]. Since the beginning of the pandemic, intensive research on the host genetic makeup has identified several genes associated with COVID-19 susceptibility and severity. Genetic susceptibility to SARS-CoV-2 infection has been associated with interferon-related genes (TLR3, IFNAR1/2, and IRF9), viral entry factors (ACE2, TMPRSS2), and specific HLA haplotypes (HLA-DRB115, HLA-A30:02) [9,10,11]. Some researchers and groups consider the major genetic determinants of severe COVID-19 to be monogenic traits [12]; however, studies performed in broad, global collaborations found several genetic traits associated with severe COVID-19 in genome-wide association studies (GWAS) [13,14]. Intriguingly, some regions of chromosome 3 that are of Neanderthal origin are associated with severe COVID-19 [15].

Genetic and genomic studies have the potential to identify strong genetic determinants that may help healthcare providers predict the course of the disease and potentially identify therapeutic targets. Research in the last 2–3 years of the COVID-19 pandemic resulted in the identification and publication of such human host genetic determinants. There are already algorithms that can predict the outcome of the disease based on comorbidities, disease symptoms, and specific genetic haplotypes [16]; however, a clear and exact gene set and polymorphism-based prediction of COVID-19 are yet to be established.

However, the strong effect of classical clinical risk factors, particularly age and comorbidities, may obscure the contribution of host genetic variation when heterogeneous patient populations are analyzed. As a result, genetic signals relevant to disease severity can remain undetected if traditional stratification strategies are applied.

The clinical course of COVID-19 is shaped by a complex interplay of demographic, clinical, and genetic factors. Established evidence indicates that age and comorbidities, such as cardiovascular disease, diabetes, or chronic pulmonary disorders, are among the strongest predictors of severe disease outcomes. However, these well-recognized determinants may mask the potential contribution of host genetic variation when analyzed in heterogeneous patient populations [4,5].

Rather than only stratifying by age and comorbidities—which are established predictors of COVID-19 severity—our approach focused on creating two contrasting groups that reflect the paradoxical nature of the disease. While younger, otherwise low-risk patients occasionally developed severe or even fatal COVID-19 [17,18], some elderly and multimorbid patients remained asymptomatic or experienced only mild disease [19,20,21]. These polar opposites formed the basis of our focus cohorts, where we hypothesized that genetic determinants underlie these unexpected outcomes. Specifically, we expected genetic factors to predispose younger patients to severe disease, while in elderly patients with unexpectedly mild disease, genetic factors were assumed to contribute to protection. To test this hypothesis, we compared the focus cohorts, in which a genetic contribution was presumed, with control cohorts in which disease outcome could be attributed primarily to age and comorbidities, consistent with classical epidemiological expectations. Although similar paradoxical patterns had been described in previous influenza outbreaks, where severe disease occasionally occurred in otherwise healthy young adults [22,23,24], and some elderly remained asymptomatic [25], these extremes became particularly prominent in COVID-19. We therefore propose that this stratification framework, originally highlighted by COVID-19, could also serve as a useful methodological approach for studying host determinants of severity in other viral infections.

From a clinical utility perspective, genetic testing is most effective when based on a limited number of carefully selected variants that can be assessed rapidly and provide meaningful predictive value. While COVID-19 is a complex, polygenic disease, our aim was not to capture the entirety of its genetic architecture but rather to establish a focused set of determinants that may be practical for diagnostic application. To this end, we combined a stringent patient stratification method with a machine learning approach to identify a concise and clinically useful gene panel.

Here, we report a newly identified set of genetic factors that are associated with the severity of COVID-19. During the study, we developed and applied a COVID-19-specific patient stratification method for Hungarian patients. These factors were identified with whole-exome sequencing (WES) and calculation of mutation load for each gene. A machine learning approach with a Random Forest algorithm was used to define the gene set that clearly distinguishes patients with respect to COVID-19 severity.

## 2. Results

### 2.1. Identification of Susceptibility and Protective Genetic Factors Responsible for COVID-19 Severity in Stratified Patient Cohorts

We conducted a WES analysis of the stratified patient cohorts using standard bioinformatics techniques, as described in the Materials and Methods section. Data were filtered, and single-nucleotide polymorphisms (SNPs) were summarized, resulting in a variant dataset of 20,048 genes (Supplementary Table S1), prior to applying machine learning.

**Figure 1:**
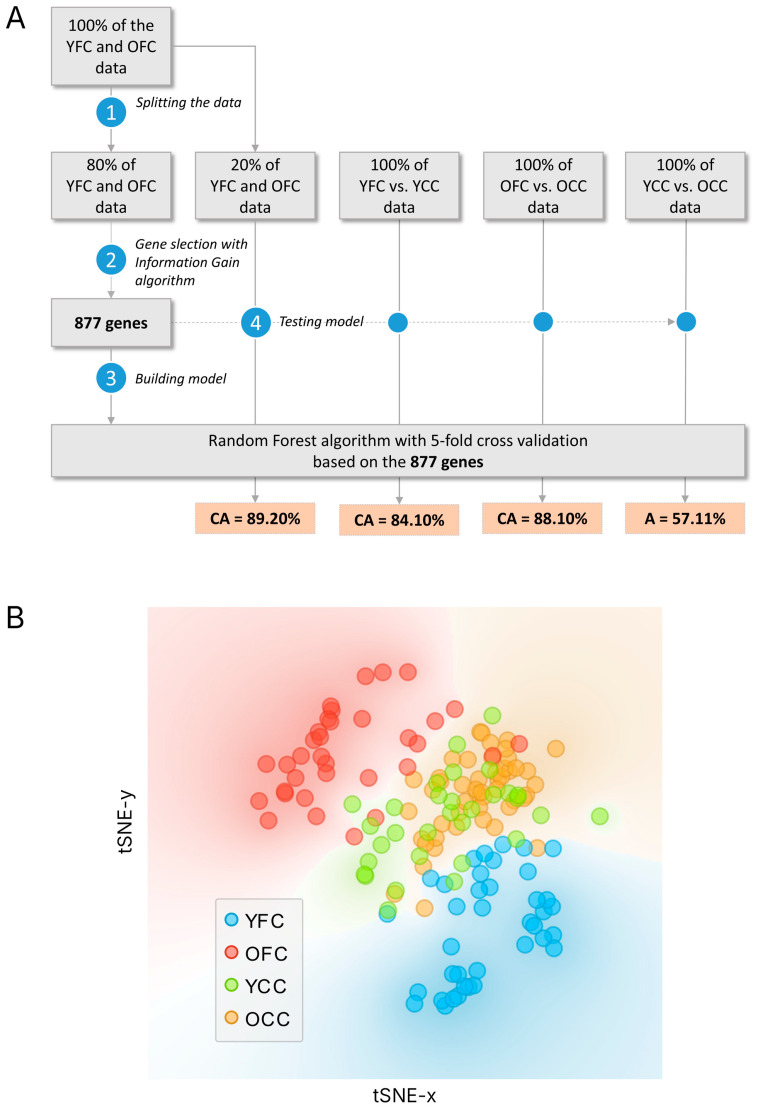
Candidate gene selection and differentiation among patient groups: (**A**) Workflow of the classification analysis. The YFC and OFC datasets were split into 80% training and 20% testing subsets. Gene selection using the information gain algorithm identified 877 genes, which were used to build a Random Forest model with 5-fold cross-validation. The model achieved a classification accuracy (CA) of 89.20% for YFC vs. OFC data. The same gene set was subsequently applied to classify YFC vs. YCC (CA = 84.10%, sensitivity = 83.80%, specificity = 84.40%), OFC vs. OCC (CA = 88.10%, sensitivity = 87.90%, specificity = 88.30%), and YCC vs. OCC (A = 57.11%, sensitivity = 56.80%, specificity = 57.40%). (**B**) t-distributed Stochastic Neighbor Embedding (t-SNE) visualization of the four patient cohorts. Note how YFC and OFC samples form distinct clusters, while control groups (YCC and OCC) overlap partially but remain separated from their corresponding focus cohorts.

For machine learning, we followed the workflow shown in Figure 1 to identify genes distinguishing the two focus groups, YFC (Young Focus Cohort, *n* = 38) and OFC (Old Focus Cohort, *n* = 34), thereby revealing host genetic factors underlying COVID-19 severity (Figure 1A).

We ran an information gain algorithm to score the genes on 80% of the data (Figure 1A, Steps 1–2). The average information gain was found to be 0.03. Genes above the mean were selected, resulting in a panel of 877 genes (Supplementary Table S2).

Subsequently, we applied the Random Forest algorithm with 5-fold cross-validation to 80% of the data, using the 877 selected genes. The model generated by the Random Forest algorithm was able to distinguish the remaining YFC and OFC data, with an average classification accuracy of 89.20% (sensitivity = 88.80%, specificity = 89.60%) (Figure 1A, Step 4).

Next, the datasets from the focus groups (YFC, OFC) were compared with their corresponding control groups, YCC (Young Control Cohort, *n* = 31) and OCC (Old Control Cohort, *n* = 49). Classification results show that, based on the mutation patterns of the 877 genes, the YFC and YCC could be distinguished with an average classification accuracy of 84.10%, corresponding to a sensitivity of 83.80% and a specificity of 84.40% (Figure 1A).

Similarly, the OFC and OCC could be separated with an accuracy of 88.10%, with a sensitivity of 87.90% and a specificity of 88.30% (Figure 1A)

In contrast, when comparing the YCC and OCC directly, the classification performance dropped to 57.11%, with a sensitivity of 56.80% and a specificity of 57.40%, indicating a substantial similarity between the two control datasets (Figure 1A).

As the final step of the analysis workflow, the dataset for the two control groups—YCC and OCC—was analyzed using the Random Forest algorithm, which employed the genetic patterns of the 877 identified genes. The method was unable to distinguish between the two control groups. This is consistent with our a priori hypothesis: differences in COVID-19 severity between the two control groups are caused mainly by classical determinants, such as age and the presence of comorbidities, not by underlying genetic factors (Figure 1A).

The final Random Forest results are shown in Figure 1B, illustrating the separation of the stratified patient cohorts (highlighted in different colors) based on the variant pattern of the 877 genes. For clarity, in the subsequent sections, we refer to genes whose variant patterns are associated with an increased likelihood of severe COVID-19 as “susceptibility genes.” In contrast, those associated with a reduced likelihood are termed “protective genes.” These designations describe statistical associations with disease outcomes rather than implying an intrinsically harmful or beneficial function of the genes themselves.

Given that YFC and OFC represent polarized severity strata, we evaluated potential confounding to ensure that the gene-based signal predicting differences between these groups reflects disease course rather than demographic or clinical covariates. Specifically, we tested whether age, sex, and major comorbidities are related to the genetic summaries and model behavior.

SNP burden was summarized per subject from the 877-gene panel and correlated with clinical variables (age, sex, major comorbidities). Across 13,155 covariate–SNP-burden pairs, only 4/13,155 associations remained significant after Benjamini–Hochberg correction (FDR q < 0.05); all corresponding effect sizes were small (|ρ| ≤ 0.50), and directions were inconsistent. The remaining 13,151/13,155 comparisons were non-significant at q ≥ 0.05, indicating that SNP burden is not meaningfully explained by demographic or comorbidity structure (Supplementary Table S3).

**Figure 2:**
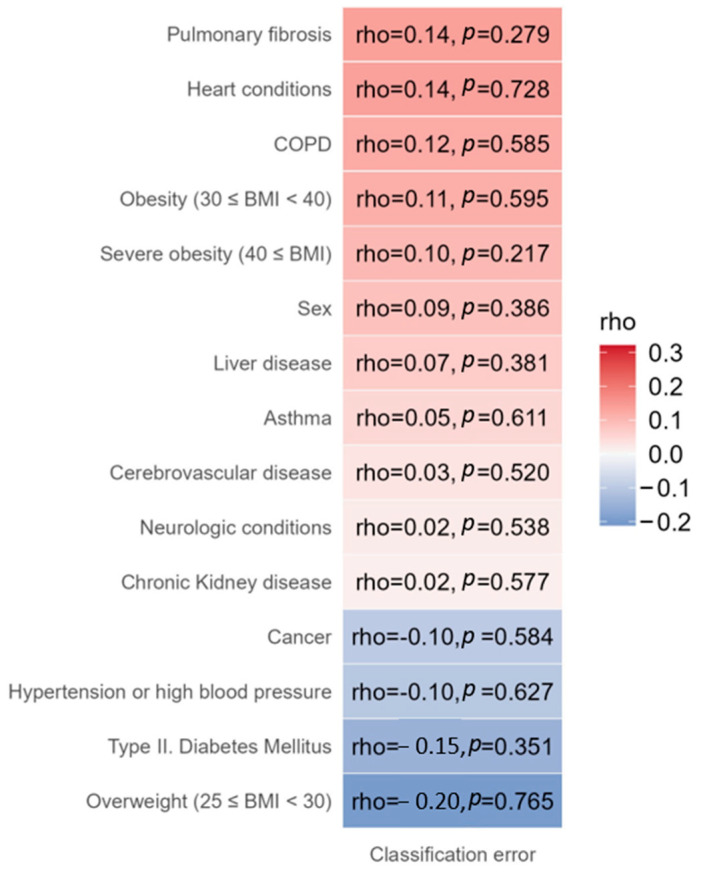
Correlations between clinical variables and per-sample classification error. Spearman’s rank correlation coefficients (rho) and corresponding *p*-values are shown for each clinical variable. All associations were weak and statistically non-significant (|ρ| = 0.02–0.20; all *p* = 0.217–0.765), indicating that model performance was not influenced by demographic or comorbidity-related factors. The color scale represents the direction and strength of correlations (red: positive; blue: negative).

To assess potential effects on model behavior, we also correlated these clinical variables with per-sample classification error. Associations were again negligible (|ρ| = -15–0.14; all *p* = 0.279–0.765; Figure 2), supporting that the classification performance reflects genetic variation rather than demographic or comorbidity profiles.

### 2.2. Genetic-Variant Landscape of the Susceptibility and Protective Genes

Within the 877 genes considered, we classified loci as susceptibility or protective by comparing mean variant counts between the two focus cohorts (YFC vs. OFC). Using this criterion, 431 genes were labeled as susceptibility genes (YFC > OFC), and 446 genes were labeled as protective (OFC ≥ YFC).

When contrasting focus groups to their age-matched controls, 246/431 susceptibility genes (57.08%) showed a higher average SNP count in OCC than in OFC, whereas 419/446 protective genes (93.95%) had a higher average SNP frequency in OFC than in OCC. In the younger cohorts, 369/431 susceptibility genes (85.61%) had higher average SNP counts in YFC than in YCC, while 302/446 protective genes (67.71%) had lower average SNP counts in YFC than in YCC.

**Figure 3:**
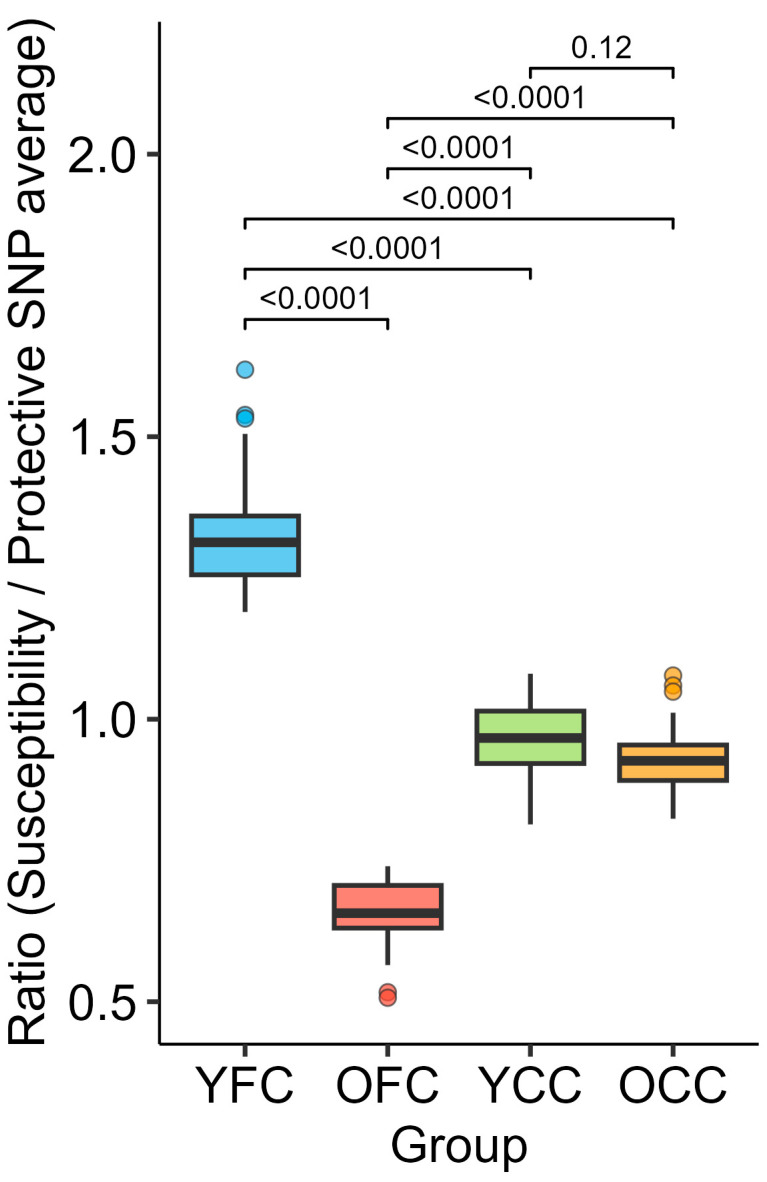
Group-wise susceptibility-to-protective SNP ratio (boxplots; one-way ANOVA *p* < 0.001). Across 877 genes, 431 were classified as susceptibility (YFC > OFC) and 446 as protective (OFC ≥ YFC). Among susceptibility genes, 246/431 (57.08%) show higher average SNP counts in OCC than OFC, and 369/431 (85.61%) are higher in YFC than YCC. Among protective genes, 419/446 (93.95%) are higher in OFC than OCC, and 302/446 (67.71%) are lower in YFC than YCC. Mean ratios: OFC 0.65, YFC 1.33, YCC 0.97, OCC 0.93—confirming that the gene-categorization directionality is preserved at the individual level.

For each individual, we computed the mean SNP count across susceptibility genes and across protective genes and formed their ratio. The resulting group means were 0.65 (OFC) and 1.33 (YFC) in the focus cohorts, and 0.97 (YCC) and 0.93 (OCC) in the controls. One-way ANOVA on these ratios revealed a strong group effect (*p* < 0.0001). Tukey *post hoc* tests indicated no significant difference between the two control groups (YCC vs. OCC: Δ = −0.04, *p* = 0.121), while both focus groups differed markedly from their corresponding controls (OFC vs. OCC: Δ = 0.27, *p* < 0.0001; YFC vs. YCC: Δ = −0.36, *p* < 0.0001) (Figure 3).

These patterns confirm that the directionality imposed by the gene categorization (YFC > OFC ⇒ susceptibility; otherwise protective) is preserved at the individual level across groups, i.e., the subject-level susceptibility-to-protective SNP ratio follows the same directionality.

### 2.3. Identification of Biological Processes Affected by Susceptibility and Protective Genes

Gene Ontology (GO) enrichment analysis of the 877 genes highlighted several significant categories (reported here as *p* values). In molecular function (MF), enrichment was observed for protein binding (GO:0005515, *p* = 0.007) and cation binding (GO:0043169, *p* = 0.02). Within the biological process (BP) category, biological regulation (GO:0065007, *p* = 0.005) was enriched. For cellular component (CC), significant terms included cytoplasm (GO:0005737, *p* = 0.007) and lateral element (GO:0000800, *p* = 0.04). Detailed results: https://biit.cs.ut.ee/gplink/l/aio83Uc8kSI (accessed on 25 September 2025).

Using the Metascape signature module (Immunological Signatures subset), the enrichment analysis of 877 genes showed a significant overrepresentation of gene sets related to macrophage activation, interferon-stimulated conditions, Toll-like receptor ligand stimulation, and T cell activation and differentiation experiments. The enriched signatures included datasets on STAT6-knockout macrophages, IFNG- and IL6-perturbed systems, and CD4^+^/CD8^+^ T cell polarization states. Complete results are provided in Supplementary Document S1.

### 2.4. Determining the Minimal Discriminating Gene Set

To enhance the practical applicability of our findings, we next sought to reduce the set of 877 genes to achieve an optimal balance between panel size and predictive efficiency.

The genes were ranked in descending order based on their information gain values, reflecting their relative contribution to the classification of YFC and OFC.

**Figure 4:**
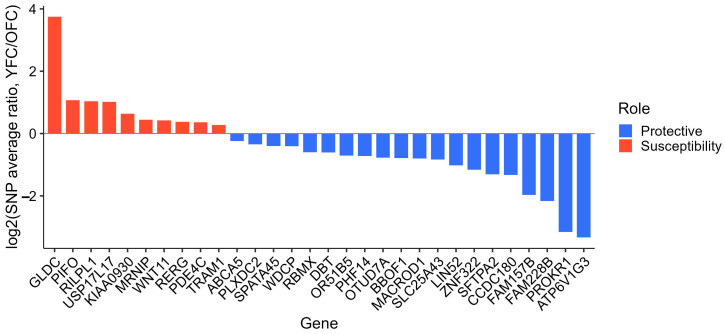
The 30 genes exerting the strongest association with disease severity. Log_2_ (severe/mild) SNP ratios for the top genes, showing susceptibility (red) and protective (blue) associations.

Subsequently, we incrementally expanded the gene panel by sequentially adding genes according to their ranked importance and evaluated the resulting models to identify the smallest subset that retained maximal classification accuracy (Figure 4).

As the number of included genes increased, classification performance initially improved, reaching a peak accuracy of 80.60% with the top 30 most significant genes (Supplementary Table S2).

**Figure 5:**
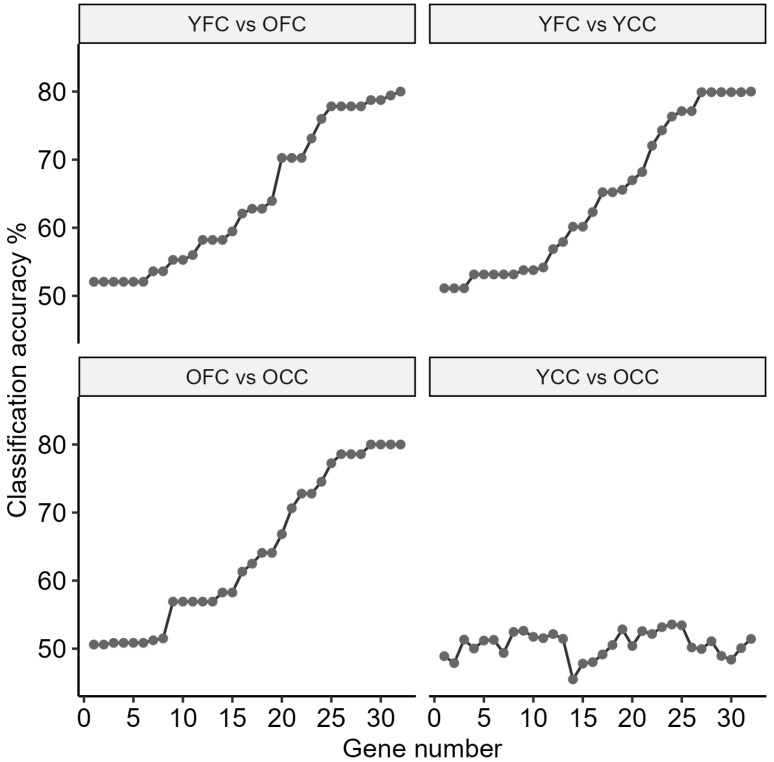
Classification accuracy as a function of the number of top-ranked genes. Each panel shows one pairwise comparison (YFC vs. OFC, YFC vs. YCC, OFC vs. OCC, and YCC vs. OCC). For each comparison, genes were ranked by information gain on the training split, and a Random Forest classifier was trained using the top *N* genes (*N* = 1–30). Points denote the mean cross-validated accuracy from 5-fold CV; lines connect points as a visual guide. Curves for YFC–OFC, YFC–YCC, and OFC–OCC rise toward ~80% as *N* increases, whereas YCC–OCC fluctuates around ~50% (chance level), indicating negligible separability of the two control cohorts.

Beyond this point, the inclusion of additional genes resulted in only minimal further improvement until the final 877-gene panel achieved the maximum accuracy observed in the model (Figure 5).

Notably, this 30-gene panel achieved an average efficiency of 90% across all comparisons. To test the predictive value of this minimal set of genes, we analyzed the control groups (YCC and OCC) and found that they did not differ from one another.

## 3. Discussion

In this study, we applied whole-exome sequencing, combined with machine learning-based feature selection, to identify genetic variants associated with COVID-19 severity in a clinically stratified Hungarian cohort. By leveraging a polarized cohort design that contrasts individuals with discordant clinical risk profiles and disease outcomes, we derived a gene panel that reflects both susceptibility- and protection-associated signals in this population. This strategy enabled the detection of variant patterns that may remain undetected in conventional severity comparisons relying solely on clinical stratification.

Several genes within the identified panel are functionally linked to biological processes previously implicated in host responses to viral infection, including immune regulation, cellular signaling, and metabolic pathways [26,27,28,29,30,31,32,33,34,35,36,37,38,39,40,41,42,43,44,45,46,47,48,49,50,51,52,53,54,55,56,57,58,59,60,61,62,63]. While these associations support the biological plausibility of the model output, it is important to emphasize that the present findings reflect statistical prioritization derived from enrichment and machine learning analyses and should not be interpreted as direct evidence of causal molecular mechanisms.

Our results show that combining a cohort design based on clinical information with exome-level variant analysis can identify candidate genetic signatures linked to disease severity. This creates a basis for future functional and translational studies. Notably, comparison with ancestry-specific severe-versus-mild analyses from the COVID-19 Host Genetics Initiative revealed no direct gene overlap with the core 30-gene panel [14,26]. This lack of concordance is not unexpected, as the final panel represents a highly constrained subset selected to optimize classification performance within our cohort, and therefore reflects a parsimonious predictive signature rather than an exhaustive catalog of disease-associated loci. In contrast, comparison at the broader feature-selection level (*n* = 877 discriminating genes) demonstrated direct overlap with several HGI-reported genes, including THBS3, FBRSL1, KANSL1, DPP9, TYK2, IFNAR2, ELF5, and SLC22A31. This pattern indicates that the analytical framework captures established host genetic signals at the broader feature-space level. In contrast, the final reduced panel prioritizes a compact subset that maximizes predictive performance within the studied cohort. Several overlapping genes converge on interferon-driven antiviral signaling and cytokine regulation, including TYK2 and IFNAR2, which directly participate in JAK–STAT-mediated immune responses [27], as well as regulators of transcriptional and inflammasome-associated signaling such as KANSL1 and DPP9 [28,29,30,31]. These links suggest a convergence in host antiviral response-modulation pathways. A second functional theme concerns epithelial–extracellular interface biology, represented by THBS3 and ELF5, which are linked to tissue organization and epithelial regulation [32,33,34,35], together with transport and epigenetic regulators such as SLC22A31 and FBRSL1 [36,37]. These associations may reflect contributions of host-tissue context to viral susceptibility and the inflammatory response.

Gene set enrichment analysis using immune signature collections revealed significant associations with transcriptional programs related to macrophage activation, interferon signaling, Toll-like receptor-mediated innate sensing, and T cell functional polarization. These processes are consistent with established host-response pathways during viral infection, where pattern-recognition receptor activation induces cytokine production and immune modulation, and interferon signaling orchestrates antiviral defense and adaptive immune coordination [38,39]. Importantly, interferon-driven signaling has been shown to regulate macrophage inflammatory responses [40] and T cell functionality during viral disease [41]. At the same time, excessive activation of innate sensing pathways may contribute to inflammatory dysregulation observed in severe COVID-19 [42,43]. Accordingly, these enrichment patterns support the biological plausibility of the identified gene set while remaining hypothesis-generating rather than indicative of causal mechanisms.

A general gene set enrichment analysis of the full discriminating gene set identified functional themes related to molecular interaction networks, metal-dependent processes, intracellular signaling environments, and epithelial organization. Instead of pointing out specific mechanisms, these enrichments offer context for biological processes that have been previously connected to host–virus interaction dynamics [44,45,46,47,48,49,50,51,52,53,54,55,56,57,58,59,60,61,62,63]. Enrichment of the protein and small-molecule binding categories highlights the role of molecular interaction networks in viral infections. Host protein–protein interfaces and cofactor-dependent processes play a part in immune signaling and the response to pathogens [44,45,46,47,48]. The representation of cation-binding functions is consistent with the involvement of metal-dependent enzymatic and regulatory pathways in cellular redox balance and antiviral defense, although these associations remain interpretive [49,50,51]. Intracellular localization signatures, especially those of cytoplasmic components, show the cellular areas where antiviral sensing, mitochondrial stress responses, and viral replication occur [52,53,54,55,56]. In parallel, enrichment for epithelial structural elements supports the relevance of tissue interface biology, including mucociliary and barrier-associated functions that are frequently implicated in respiratory disease severity [57,58,59]. Finally, broad regulatory process categories indicate that prioritized genes span diverse roles in transcriptional, metabolic, and cell cycle regulation [60,61,62,63]. Taken together, these pathway-level observations suggest functional convergence across interaction networks, cellular regulation, and tissue interface processes; however, they should be interpreted as hypothesis-generating annotations rather than evidence of causal mechanistic involvement.

Monogenic [64,65] and polygenic-focused [13,66,67,68] genetic studies provide complementary approaches to understanding the role of host genetics in COVID-19 susceptibility and severity. While the two approaches differ in methodology and scope, they can provide valuable insights when combined. Broad analyses of genetic data can help identify common genetic variants influencing COVID-19 outcomes across populations. In contrast, research on rare genetic mutations provides specific insights into how individual immune responses affect disease severity.

Lai et al. (2022) and Zheng et al. (2024) both used transcriptomic data and bioinformatics approaches to study COVID-19, with Lai et al. constructing a three-gene diagnostic signature and Zheng et al. developing an immune cell infiltration (ICI) score to classify immune subtypes and predict severity [69,70]. While such transcriptomic-based tools capture the dynamic immune landscape and reflect disease severity during infection, our study takes a fundamentally different approach by focusing on underlying genetic predisposition. Using WES in a well-defined Hungarian cohort and a stratification method based on age, background, and disease severity, we combined mutational load analysis with a Random Forest model to identify genetic variants associated with protection against or susceptibility to severe COVID-19. In contrast to expression-based methods, our SNP-based severity panel uncovers host-level genomic determinants and can identify individuals at risk before or early in infection. Together, these complementary approaches could provide a multi-layered framework for risk prediction and patient stratification.

The present findings provide a foundation for several ongoing and planned research directions aimed at refining biological interpretation and translational relevance. Future work will focus on the functional characterization of prioritized genes through gene-expression profiling, in vitro assays, and targeted mechanistic studies, including pathway-focused perturbation approaches.

Additional validation efforts will assess the strength and applicability of the predictive framework in independent cohorts. This will help determine how the proposed gene panel can be used for clinical classification. We also plan to conduct comparative analyses across different respiratory diseases, such as influenza. This will allow us to explore whether the identified signals are specific to COVID-19 or reflect broader patterns of host susceptibility. We are currently conducting complementary transcriptomic profiling of samples from the same cohort. This enables combined genomic and expression analyses, which may reveal downstream regulatory patterns and guide subsequent experimental validation. Together, these efforts aim to advance the interpretation of statistically prioritized signals toward biologically grounded and clinically informative applications.

### Strengths and Limitations

This pilot study employs WES and machine learning to examine genetic variants associated with COVID-19 severity in a Central-Eastern European (Hungarian) cohort, which is largely underrepresented in current genomic research. A major strength of our approach is the clinical–genetic stratification of patients. This offers a useful framework for future precision risk assessments. Using machine learning helped us identify candidate genes that may predict disease severity. This shows the potential of data-driven methods in infectious disease genomics. Our methodology could serve as a proof of concept for including genomic data in clinical decision-making processes. Nonetheless, several limitations should be acknowledged. The modest sample size and the single-population design may limit the generalizability of our findings, although the inclusion of an underrepresented ancestry also enhances the diversity of existing datasets. Functional validation of the implicated genes was beyond the scope of this study; therefore, the reported associations should not be interpreted as direct evidence of causal biological mechanisms. Further work will be required to confirm these findings through gene expression analyses and mechanistic studies.

Furthermore, while certain well-known immune-related genes, such as those in the interferon signaling pathway, were not prioritized by the model, their biological relevance cannot be excluded. It will be further evaluated in future panel iterations.

Despite cross-validation, using a single dataset for both feature selection and model training introduces the risk of overfitting and may overestimate predictive performance. This highlights the importance of validating the proposed gene panel and machine learning framework in independent external cohorts. Future validation of the proposed 30-gene panel should involve its application to independent external cohorts using the same stratification criteria and machine learning framework, followed by an evaluation of predictive performance and reproducibility across different populations.

Overall, the study provides an important first step toward establishing a genetically informed framework for COVID-19 severity stratification and lays the groundwork for expanded, multi-ethnic validation and functional follow-up.

## 4. Materials and Methods

### 4.1. Patient Recruitment

This study was conducted at the Albert Szent-Györgyi Clinical Centre of the University of Szeged and the University of Pécs, First Department of Internal Medicine. From March 2021 to December 2022, after applying strict and unique enrollment criteria (described in detail in Materials and Methods, Section 4.3) a total of 700 patients were enrolled in the study. For the prospective part of the study, patients over 18 years of age with positive COVID-19 PCR tests, which were not older than 48 h, were recruited. COVID-19 positivity was confirmed using the SARS-CoV-2 real-time PCR assay on nasopharyngeal samples. After the patient signed the informed consent sheet, relevant patient data were collected, including medical history and demographic data. Biological samples (peripheral blood and nasopharyngeal swabs) were collected according to the ethical approval (19697-6/2020/EÜIG) issued by the National Center for Public Health, Hungary.

### 4.2. Development of a Unique Patient Stratification Method

The disease severity of the patients was determined based on the instructions in the Hungarian COVID-19 manual [71]. Patients were classified into five groups (asymptomatic, mild, moderate, severe, and critical COVID-19) according to the most severe stage of their disease progression.

For the basis of our genetic study, we developed a unique and stringent stratification method. Age was one of the bases of our distinction between the cohorts. According to the calculations by the Centers for Disease Control and Prevention (CDC, cdc.gov) and as reported in the international literature, the course of COVID-19 was more severe in patients over 65 years of age than in younger individuals [72]. Thus, we chose this age as a cut-off for specifying patient groups. Other distinguishing aspects were clinical risk factors for severe COVID-19 identified in epidemiological studies [72,73,74,75,76,77,78,79,80,81,82,83,84,85,86,87,88,89,90,91,92,93,94]. We defined four patient groups based on age, COVID-19 severity, and the presence of major and minor risk factors. Patients were assigned to one of four pre-defined cohorts immediately after their enrollment. The definition of these patient groups ensured homogeneity within groups and a clear separation between the focus and control cohorts (genetic determinants of severity vs. environmental determinants of severity).

For this separation, clinical data were collected from all enrolled patients based on the risk stratification criteria issued by the CDC at the beginning of the COVID-19 pandemic. Data collection initially encompassed major and minor clinical risk factors for severe COVID-19, including chronic cardiovascular, pulmonary, renal, metabolic, oncological, and neurological conditions, obesity-related categories, smoking status, pregnancy, and immunocompromised states.

As evidence accumulated, the stratification framework was iteratively refined to reflect emerging, new epidemiological data. Patients with thalassemia and sickle cell disease were excluded, as their independent associations with COVID-19 severity remained inconclusive. Individuals with immunocompromised states were also excluded to avoid confounding effects on disease severity and immune response. In addition, risk factors with no representation in the cohort—namely Down syndrome, pregnancy, cystic fibrosis, and type 1 diabetes mellitus—were removed from the scoring system. These refinements resulted in a streamlined and unique stratification approach suitable for genetic analyses. The complete list of risk factors and a detailed table of the reasoning behind patient cohorts can be found in Supplementary Document S2 [95].

### 4.3. Cohort Definitions

Based on the predefined stratification framework, we specified four distinct patient cohorts, which allowed us to map the contribution of genetic versus clinical factors to COVID-19 outcomes. The young focus cohort (YFC, *n* = 38) comprised patients under 65 years of age who developed severe or critical COVID-19 (severity categories 4–5) despite having few or no clinical risk factors. In contrast, the old focus cohort (OFC, *n* = 34) included patients over 65 years presenting with only mild-to-moderate symptoms (severity categories 1–2), in the presence of multiple comorbidities. These two groups were designed to highlight outlier cases where disease severity could not be explained by age or comorbidities alone.

For comparison, we also defined two control cohorts, representing more typical clinical outcomes. The young control cohort (YCC, *n* = 31) consisted of patients under 65 years with mild COVID-19 (severity categories 1–2) and minimal comorbidities, while the old control cohort (OCC, *n* = 49) included older patients (≥65 years) with severe disease (severity categories 4–5) and a high burden of risk factors. Together, these four cohorts provided a structured framework to contrast genetic susceptibility with severity, which contributes to well-known clinical determinants. In Table 1, we provide a general description of our patient cohorts.

**Figure 6:**
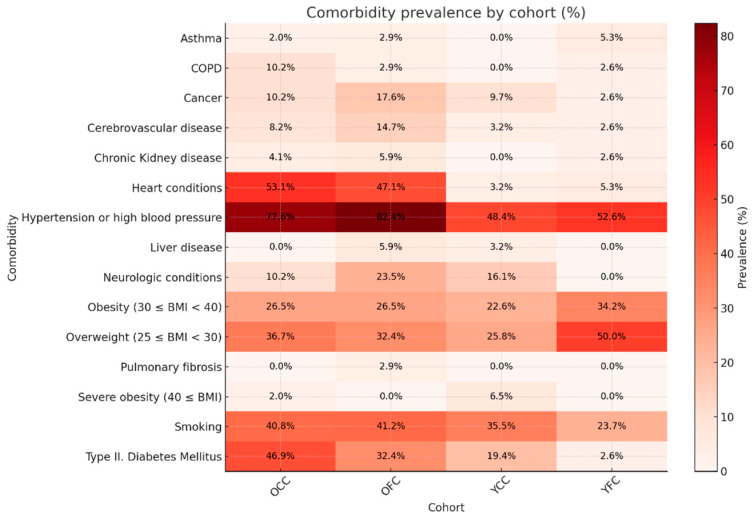
Heatmap representation of risk factor prevalence per study cohort. The heatmap shows the percentage of patients affected by each comorbidity within the four cohorts. Warmer colors indicate a higher prevalence. As expected, comorbidities were more frequent in the older cohorts (OFC, OCC), while the young cohorts (YFC, YCC) displayed markedly lower rates.

The prevalence of each risk factor in every cohort can be seen in Figure 6.

**Figure 7:**
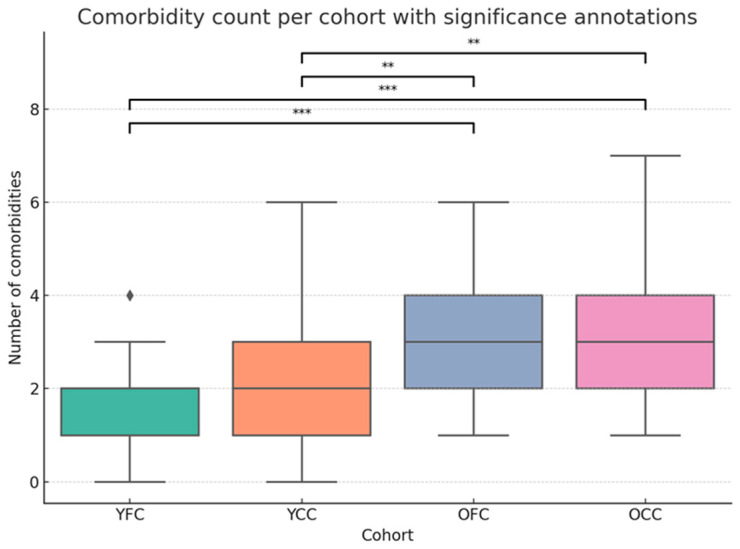
Comorbidity counts across patient cohorts. Boxplots show the distribution of comorbidity counts in the young focus cohort (YFC), young control cohort (YCC), old focus cohort (OFC), and old control cohort (OCC). Older cohorts displayed significantly higher comorbidity counts than younger ones, while no difference was found between OFC and OCC or between YFC and YCC. Significance was determined by pairwise Mann–Whitney U tests with Bonferroni correction (*** *p* < 0.001, ** *p* < 0.01).

We tested whether our a priori patient stratification strategy resulted in comparable patient cohorts. The distribution of risk factors per cohort can be found in Figure 7.

To assess whether the patient cohorts differed in overall comorbidity burden, we compared the number of comorbidities per patient across groups. The Kruskal–Wallis test indicated a significant difference between cohorts (χ^2^(3) = 36.42, *p* < 0.001). Post hoc pairwise Mann–Whitney U tests with Bonferroni correction revealed that the young cohorts (YFC, YCC) did not differ from each other, and likewise, no difference was detected between the two older cohorts (OFC, OCC). In contrast, all comparisons between young versus older cohorts were significant (*p* < 0.001), confirming that the stratification effectively separated patients with low versus high comorbidity burden.

### 4.4. DNA Extraction

Blood samples were taken from the patients, and DNA was extracted from the frozen, EDTA-treated samples using the QIAsymphony DSP DNA Kit (QIAGEN, Hilden, Germany) with the help of the QIAsymphony SP nucleic acid purification instrument, according to the manufacturer’s instructions.

### 4.5. Whole-Exome Sequencing

Whole-exome libraries were prepared using the DNA Prep with Enrichment workflow (Illumina, Inc., San Diego, CA, USA). Briefly, 100 ng of genomic DNA was fragmented, amplified, and purified. Exome capture was performed using Illumina Exome Panel probes (Illumina). The captured libraries were quality-checked using TapeStation 4200 (Agilent Technologies, Santa Clara, CA, USA) and Qubit 3.0 (Thermo Fisher Scientific, Waltham, MA, USA). The libraries were sequenced on NovaSeq 6000 (Illumina) for 2 × 150 paired-end reads.

### 4.6. Bioinformatics Analysis

Raw sequence generation, including base calling and demultiplexing, was performed using bcl2fastq (v2.20.0.422) (bcl2fastq (RRID: SCR_015058)) on a local high-performance computing (HPC) cluster. First, the quality of the raw FASTQ files was checked by FastQC (v0.11.9) [96]. Based on the quality control results, the dataset was filtered, and adapters and low-quality bases/reads were removed with fastp (v0.21.0) [97]. Clean, high-quality reads were aligned to the human reference genome (GRCh37) by applying the bwa mem algorithm with default parameters (v0.7.17) [98]. BAM file modifications (e.g., sorting, adding read group information, indexing, and deduplication) were performed using Picard Tools (v2.23.3) subcommands (http://broadinstitute.github.io/picard/ accessed on 26 September 2025). Mapping statistics and coverage metrics were calculated and collected by Picard Tools (v2.23.3) and Qualimap (v2.2.1) bamqc [99], respectively. Before variant calling, base quality scores were recalibrated by the GATK BSQR module (v4.5.0.0). Single-nucleotide variants (SNVs) and short insertions and deletions (INDELs) were identified with the GATK HaplotypeCaller algorithm by applying +/− 100 bp padding to the target region. Individual samples were merged into a joint VCF to perform the VQSR step for SNPs and INDELs by applying GATK CombineGVCFs, VariantRecalibrator, and ApplyVQSR modules (truth sensitivity filter level 99.5 for SNPs and 99.0 for INDELs) using dbsnp156, hg38-v0-1000G_omni2.5, and hg38-v0-1000G_phase1.snps, hapmap_3.3.hg38 and Mills_and_1000G_gold_standard.indels databases (accessed on 26 September 2025). Next, raw variants were pre-filtered by the GATK VariantFiltration module [100]. For SNPs and INDELs, the “QD < 2.0 || FS > 60.0 || MQ < 40.0” and “QD < 2.0 || FS > || 200.0 || GQ < 20 || DP < 19” parameters were used, respectively. Finally, variants with the “PASS” flag were selected and were functionally annotated using SnpEff v5.1b [101] to match variants with genes, and ANNOVAR (Version: 2018-04-16) was applied for further annotation using ClinVar (2025.07.29) [102]. For downstream analyses, variants with LOW annotation impact (from SnpEff), protective, conflicting, benign, and uncertain significance (from ClinVar) tags were filtered out as well. Since the average number of INDELs was significantly decreased after the filtering steps, only high-confidence SNPs were used in the downstream analyses. SNPs were summed up per gene and per sample in a matrix using R v4.5.1 (https://www.R-project.org/, accessed on 26 September 2025). The mutation matrix was used as input to compare mutation load among the predefined groups (YFC, OFC, YCC, and OCC) for each gene using the previously described machine learning data analysis.

### 4.7. Machine Learning and Data Analysis

The analyses were performed with Orange 3.35.0 software. For feature selection, we used an information gain algorithm with the Rank package. The visualization based on the selected features was performed using t-distributed Stochastic Neighbor Embedding (t-SNE), applying Global Structure Preservation, Data Scaling, and Principal Component Analysis preprocessing, which are available within the package. The classification was performed using the Random Forest algorithm based on the selected features. This involved creating 500 classification trees using the square root of the number of features at each split. The efficiency of the classification was expressed in terms of sensitivity, specificity, and classification accuracy. Gene set enrichment analysis was performed using the Reactome Pathway Database (v.84). In some cases, the continuous variables between groups were compared using one-way ANOVA with Tukey’s post hoc test. In all instances, *p* < 0.05 was considered significant.

### 4.8. Gene Set Enrichment Analysis

The gene set enrichment analysis was performed simultaneously on the 494 genes, with the statistical domain scope set to all annotated genes. Multiple testing correction was carried out using the g:SCS algorithm, which was specifically developed for GSEA analysis. We used the following online tool: https://biit.cs.ut.ee/gplink/l/aio83Uc8kSI (accessed on 25 September 2025).

## 5. Conclusions

Here, we report 30 genes that were the minimum set of identified susceptibility and protective genes for the precise and accurate prediction of disease severity in the investigated Hungarian patient cohort. Assessing the risk of whether an individual will develop severe COVID-19 is important for the patient and the clinicians involved in the treatment. Genetic screening with the identified 30 genes could potentially provide a cost-effective approach, especially in light of the decreasing costs of WES and screening based on gene panels. Further work is necessary to determine if the identified genes can aid in precise prediction in other populations. The use of this minimal gene set could aid the prediction of COVID-19 disease severity, which could have an important impact on treatment decisions. Our results could also contribute to a better understanding of the pathomechanism of the disease, elucidating molecular pathways involved in COVID-19 disease development, possibly revealing novel therapeutic targets and modalities.

## Figures and Tables

**Table 1:** Descriptive summary of the patient cohorts.

Cohort	N	Mean Age	Median Age	SD Age	Male (N)	Female (N)	Mean Severity	Median Severity
OCC	49	75.184	75	7.126	28	19	4.326	4
OFC	34	75.735	74	7.805	12	21	1.853	2
YCC	31	49.774	53	10.698	14	17	1.806	2
YFC	38	54.131	56	8.302	25	12	4.131	4

## Data Availability

The datasets used and/or analyzed during the current study are available from the corresponding author upon reasonable request.

## Supplementary Materials

The following supporting information can be downloaded at: https://www.mdpi.com/article/10.3390/ijms27052358/s1.

## Abbreviations

The following abbreviations are used in this manuscript:

COVID-19	Coronavirus disease 2019
GWAS	Genome-Wide Association Studies
WES	Whole-exome sequencing
SARS-CoV-2	Severe acute respiratory syndrome coronavirus 2
YFC	Young focus cohort
OFC	Old focus cohort
YCC	Young control cohort
OCC	Old control cohort
DNA	Deoxyribonucleic acid
EDTA	Ethylenediaminetetraacetic acid
ANOVA	Analysis of variance
HPC	High-performance computing
BAM	Binary alignment and map
GATK	Genome Analysis Toolkit
BSQR	Base quality score recalibration
SNV	Single-nucleotide variant
INDEL	Insertion/deletion polymorphism
GSEA	Gene set enrichment analysis
t-SNE	t-distributed stochastic neighbor embedding
CDK	Cyclin-dependent Kinase
PBMCs	Peripheral blood mononuclear cells
