# Supplementary material for: A Unique Patient Stratification Method Combined with a Machine Learning Approach Identifies Novel Genetic Susceptibility and Protective Factors for Severe COVID-19 in a Hungarian Population
Source: Int J Mol Sci. 2026 Mar 3;27(5):2358. doi: 10.3390/ijms27052358 (PMC12986284; doi:10.3390/ijms27052358)
Supplement: Supplementary file 1 [file ijms-27-02358-s001.zip › Supplementary_Document_S1.pdf]

Results of the g:Profiler analysis

source	term_name	term_id	highlighted	adjusted p_value	negative_log10 of adjusted p_value	term_size	query_size	intersection_size	effective domain_size
GO:MF	protein binding	GO:0005515	true	0,0038	2,4166	15123	738	601	20246
GO:MF	cation binding	GO:0043169	true	0,0278	1,5562	4495	738	209	20246
GO:MF	metal ion binding	GO:0046872	false	0,0405	1,3923	4396	738	204	20246
GO:BP	biological regulation	GO:0065007	true	0,0046	2,3384	12743	687	474	21026
GO:BP	positive regulation of biological process	GO:0048518	false	0,0083	2,0811	6264	687	259	21026
GO:BP	regulation of cellular process	GO:0050794	false	0,0290	1,5383	11946	687	444	21026
GO:BP	regulation of biological process	GO:0050789	false	0,0326	1,4872	12336	687	456	21026
GO:CC	cytoplasm	GO:0005737	true	0,0114	1,9440	12527	734	468	22149
GO:CC	lateral element	GO:0000800	true	0,0447	1,3494	16	734	5	22149

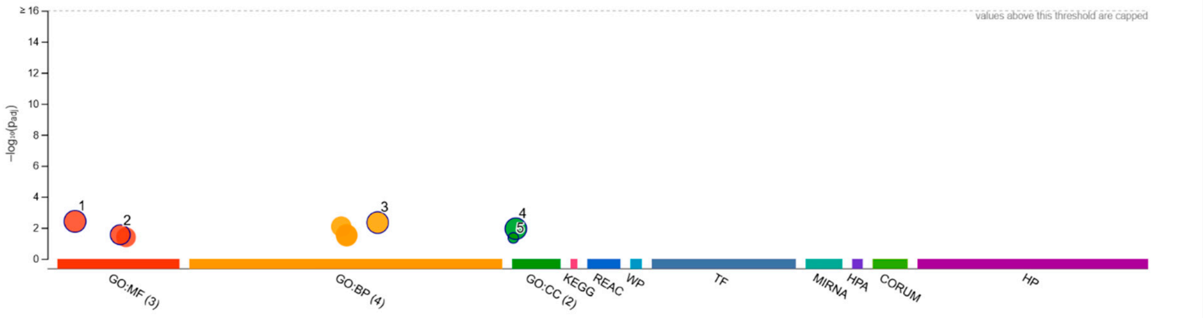

ID	Source	Term ID	Term Name	p_{adj} (query_1)
1	GO:MF	GO:0005515	protein binding	3.832×10^{-3}
2	GO:MF	GO:0043169	cation binding	2.778×10^{-2}
3	GO:BP	GO:0065007	biological regulation	4.588×10^{-3}
4	GO:CC	GO:0005737	cytoplasm	1.138×10^{-2}
5	GO:CC	GO:0000800	lateral element	4.473×10^{-2}

**version**	e113_eg59_p19_6be52918
**date**	2026. 02. 04. 12:50:17
**organism**	hsapiens

g:Profiler

Enrichment analysis using g:Profiler revealed significant Gene Ontology terms in MF, BP, and CC categories, including protein binding (GO:0005515), cation binding (GO:0043169), biological regulation (GO:0065007), cytoplasm (GO:0005737), and lateral element (GO:0000800) (adjusted $p < 0.05$).

Results of the Metascape Immunological signatures analysis

The 877 genes were analyzed by https://metascape.org/ .

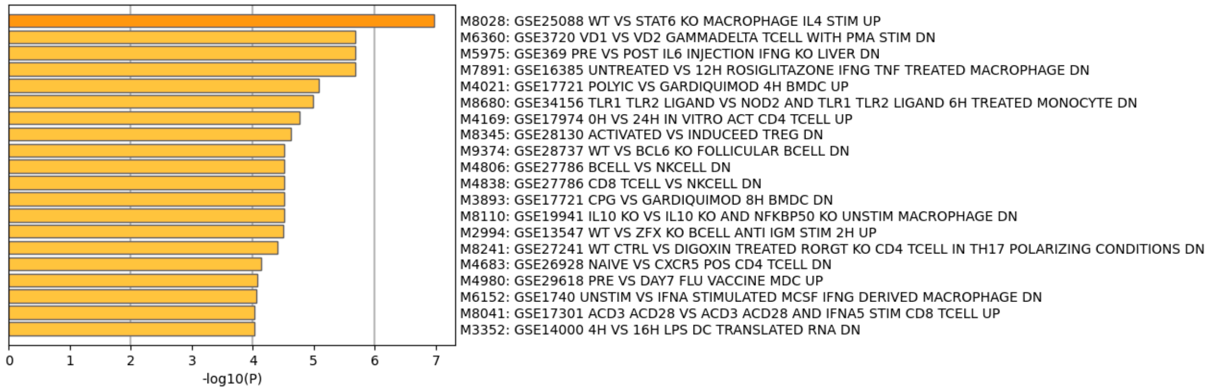

Immunologic signature enrichment of the 877-gene set.

Immunologic signature enrichment analysis was performed using the Metascape signature module with the Immunological Signatures (C7) subset of MSigDB. The bar plot shows the top enriched immune-related gene sets derived from experimental macrophage and T cell activation and cytokine-stimulation conditions. Bars represent the −log10(P) values for each enriched signature. Only significantly enriched signatures (adjusted $p < 0.05$) are shown.
