# Supplementary material for: A Unique Patient Stratification Method Combined with a Machine Learning Approach Identifies Novel Genetic Susceptibility and Protective Factors for Severe COVID-19 in a Hungarian Population
Source: Int J Mol Sci. 2026 Mar 3;27(5):2358. doi: 10.3390/ijms27052358 (PMC12986284; doi:10.3390/ijms27052358)
Supplement: Supplementary file 1 [file ijms-27-02358-s001.zip › Supplementary_Document_S2.pdf]

Supplementary Document 2.

Rationale for patient stratification

Group label	Age group	Clinical risk profile	COVID-19 severity category	Observed disease course	Primary determinant	Rationale for classification
**YFC** (Young Focus Cohort)	18-65	Low-risk	Severe (4–5)	Disproportionately severe	**Genetic**	Young patients with minimal known clinical risk factors who developed severe COVID-19, suggesting a predominant genetic susceptibility
**OFC** (Old Focus Cohort)	≥66	High-risk	Mild (1–2)	Disproportionately mild	**Genetic**	Elderly patients with multiple risk factors who experienced mild disease, indicating the presence of protective genetic factors
**YCC** (Young Control Cohort)	18-65	Low-risk	Mild (1–2)	Expected mild	**Environmental**	Young, low-risk patients with mild disease, consistent with expected clinical outcome and minimal genetic contribution
**OCC** (Old Control Cohort)	≥66	High-risk	Severe (4–5)	Expected severe	**Environmental**	Elderly, high-risk patients with severe disease, consistent with established clinical and environmental risk factors

Risk factor analysis for patient stratification

We collected the following relevant clinical data from the enrolled patients based on guidelines issued by the CDC at the beginning of the pandemic:

Major risk factors:

- Cancer
- Chronic kidney disease
- COPD
- Down syndrome
- Heart conditions
- Immunocompromised state (solid organ transplant)
- Obesity (BMI 30–39.9 kg/m²)
- Severe obesity (BMI ≥40 kg/m²)
- Pregnancy
- Sickle cell disease

- Smoking
- Type II diabetes mellitus

Minor risk factors:

- Asthma
- Cerebrovascular disease
- Cystic fibrosis
- Hypertension (high blood pressure)
- Immunocompromised state (bone marrow transplant, immune deficiencies, HIV, corticosteroids)
- Neurologic conditions
- Liver disease
- Overweight (BMI 25–29.9 kg/m²)
- Pulmonary fibrosis
- Thalassemia
- Type I diabetes mellitus

As evidence accumulated, patients with thalassemia and sickle cell disease were excluded from the scoring system due to the currently inconclusive association with COVID-19 severity. We also excluded patients with an immunocompromised state to prevent the possibility of bias in disease severity. Due to underrepresentation in the patient cohort, we also removed risk factors with 0 patients (Down syndrome, Pregnancy, Cystic fibrosis and Type I diabetes mellitus).

The patients in the Young Focus Cohort (YFC) are under the age of 65, and their COVID-19 course was severe or critical. They have up to two minor risk factors and no major risk factors. In the Old Focus Cohort (OFC), the patients are over the age of 65, and the COVID-19 course was asymptomatic or mild. They have at least two minor risk factors and one major risk factor. The patients in the Young Control Cohort (YCC) are under the age of 65, and their COVID-19 symptoms were minor or asymptomatic. The patients in the Old Control Cohort (OCC) are over the age of 65 and had a severe or critical COVID-19 course.

Clinical diagnoses corresponding to each risk factor were classified according to the International Statistical Classification of Diseases and Related Health Problems, 10th Revision (ICD-10) [1].

References:

1. World Health Organization. International Statistical Classification of Diseases and Related Health Problems, 10th Revision (ICD-10), 2nd ed.; WHO Press: Geneva, Switzerland, 2004. https://icd.who.int/browse10/2019/en
