# Supplementary material for: A Unique Patient Stratification Method Combined with a Machine Learning Approach Identifies Novel Genetic Susceptibility and Protective Factors for Severe COVID-19 in a Hungarian Population
Source: Int J Mol Sci. 2026 Mar 3;27(5):2358. doi: 10.3390/ijms27052358 (PMC12986284; doi:10.3390/ijms27052358)
Supplement: Supplementary file 1 [file ijms-27-02358-s001.zip › Supplementary_Document_S3.pdf]

Members of the COVID-19 HUNGEN research team:

Dr. Bende Balázs

Dr. Pál Margit

Dr. Bokor Barbara

Dr. Blazovits Zsófia

Dr. Veréb Zoltán

Dr. Berkecz-Kovács Lívia

Dr. Visnyovszky Ádám

Dr. Pannonhalmi Ádám

Dr. Pető Zoltán

Dr. Hajdú Edit

Dr. Farkas Dóra

Dr. Diósi Áron

Neller Alexandra

Bukva Mátyás

Dr. Burián Katalin

Dr. Somogyvári Ferenc

Dr. Endrész Valéria

Pappné Dr. Terhes Gabriella

Kispéterné Gál Mónika

Vigyikánné Váradi Anikó

Bognár Renáta

Urbán Szilvia

Dr. Varga Szilvia

Dr. Bedekovics Nikolett

Dr. Újházi Mihály

Dr. Dobos Nikoletta Alexandra

Katona-Forrai Dorina

Dr. Nagy Nikolett

Prof. Dr. Kovács L. Gábor

Dr. Gyenesei Attila

Dr. Péterfi Zoltán

Dr. Gombos Katalin

Berta Zsófia

Urbán Péter

Czuni Lilla Adrienn

Dr. Gálik Bence

Madarász Réka

Dr. Madarassi-Papp Edit

Dr. Sarlós Patrícia

Levang Szilvia

Dimák Adrienn

Herczeg Róbert

Végh Tamara

The complete list of participants in the COVID-19 HUNGEN project.
